# mHealth Technologies for Managing Problematic Pornography Use: Content Analysis

**DOI:** 10.2196/39869

**Published:** 2022-10-13

**Authors:** Nathan Henry, Liesje Donkin, Matt Williams, Mangor Pedersen

**Affiliations:** 1 Auckland University of Technology Auckland New Zealand; 2 Massey University Auckland New Zealand

**Keywords:** pornography, compulsive sexual behavior disorder, CSBD, mobile health, mHealth, problematic pornography use, PPU, mobile intervention, just-in-time adaptive intervention, smartphone-based therapy, addiction, internet addiction, behavioral addiction, mobile phone

## Abstract

**Background:**

Several mobile apps are currently available that purportedly help with managing *pornography addiction*. However, the utility of these apps is unclear, given the lack of literature on the effectiveness of mobile health solutions for problematic pornography use. Little is also known about the content, structure, and features of these apps.

**Objective:**

This study aims to characterize the purpose, content, and popularity of mobile apps that claim to manage pornography addiction.

**Methods:**

The phrase “pornography addiction” was entered as a search term in the app stores of the two major mobile phone platforms (Android and iOS). App features were categorized according to a coding scheme that contained 16 categories. Apps were included in the analysis if they were described as helpful for reducing pornography use, and data were extracted from the store descriptions of the apps. Metrics such as number of user ratings, mean rating score, and number of installations were analyzed on a per-feature basis.

**Results:**

In total, 170 apps from both app stores met the inclusion criteria. The five most common and popular features, both in terms of number of apps with each feature and minimum possible number of installations, were the ability to track the time since last relapse (apps with feature=72/170, 42.4%; minimum possible number of installations=6,388,000), tutorials and coaching (apps with feature=63/170, 37.1%; minimum possible number of installations=9,286,505), access to accountability partners or communities (apps with feature=51/170, 30%; minimum possible number of installations=5,544,500), content blocking or content monitoring (apps with feature=46/170, 27.1%; minimum possible number of installations=17,883,000), and a reward system for progress (apps with feature=34/170, 20%; minimum possible number of installations=4,425,300). Of these features, content-blocking apps had the highest minimum possible number of installations. Content blocking was also the most detected feature combination in a combinatorial analysis (with 28 apps having only this feature), but it also had the lowest mean consumer satisfaction rating (4.04) and second-lowest median rating (4.00) out of 5 stars. None of the apps reviewed contained references to literature that provided direct evidence for the app’s efficacy or safety.

**Conclusions:**

There are several apps with the potential to provide low- or zero-cost real-time interventions for people struggling to manage problematic pornography use. Popular app features include blockers of pornographic content, behavior monitoring, and tutorials that instruct users how to eliminate pornography use. However, there is currently no empirical evidence to support the effectiveness and safety of these apps. Further research is required to be able to provide recommendations about which apps (and app features) are safe for public consumption.

## Introduction

### Background

Internet pornography is becoming increasingly normalized worldwide because the rapid proliferation of the internet and personal computing devices, coupled with widespread cultural acceptance of pornography, has accelerated its spread [1]. An analysis of website traffic in Poland found a 310% increase in the number of people who used pornography on the web between October 2004 (2.76 million) and October 2016 (8.54 million) [2]. In 2019 alone, there were >42 billion visits to Pornhub, the world’s most popular pornography website [3]. The use of the internet for sexual pleasure is likely to become more prevalent as people’s time spent on the internet increases [4].

Problematic pornography use (PPU)—defined here as compulsive, dysregulated, or excessive pornography use—is considered a subcategory of compulsive sexual behavior disorder in the International Classification of Diseases, Eleventh Revision. However, there remains controversy over whether *pornography addiction* in the classical sense of an addiction exists, with opponents of this construct suggesting that compulsive pornography use is a coping mechanism for issues such as depression and anxiety. Still, there remains a high prevalence of anecdotal evidence for self-perceived pornography addiction in popular culture and web-based discourse, with many believing that pornography use can cause both depression and anxiety, among other disorders [5].

### Technological Interventions for PPU

The introduction of smartphones into modern society provides an unprecedented opportunity to deliver mobile therapies to people on a global scale. A mobile app that can anonymously guide the user through the steps required to manage PPU or regulate internet content could be valuable to people who are uncomfortable disclosing their struggle with PPU to another person. Such mobile health (mHealth) apps already exist, and unlike traditional therapy, these apps are often free and immediately available, thus supporting user autonomy and expanding access. However, it should be noted that such interventions are often hindered by low treatment commitment and adherence, limited flexibility and tailoring, and higher dropout rates than in-person therapies [6,7].

In a qualitative review of abstinence journals on a web-based *rebooting* forum for PPU, Fernandez et al [8] found that many of the forum’s members struggled with “the seeming inescapability of cues” that triggered pornography cravings, particularly on electronic media such as the internet or television. Furthermore, these cues often appear unpredictably, making casual internet browsing a risky behavior for those attempting to reduce PPU [4,8-11]. Before internet pornography was introduced, one simply had to distance oneself from physical sources of pornography, such as magazines [12]. Today, distancing is harder to achieve, given the amount of time we are connected to the internet and the ready accessibility of web-based pornography through our devices.

### Currently Available mHealth Therapies

Despite the popularity and potential of mHealth apps for managing PPU [13], there remains practically no literature assessing their effectiveness. This is consistent with the lack of literature on many other categories of mHealth apps, such as those for nonmedical cannabis use, mindfulness, and posttraumatic stress disorder, all of which have a large variety of apps available in both Google’s and Apple’s app ecosystems [14-16]. This is concerning because it makes it difficult to determine whether people are receiving evidence-based or effective interventions. Colbert et al [17] reviewed studies of 19 mobile apps designed to manage alcohol consumption; of these apps, only 8 were available in public app stores, and only 4 of these 8 apps had been demonstrated to help reduce alcohol consumption in the literature. A similarly low conversion rate has been observed in related fields [18].

One example of a therapeutic technique for PPU management is an app that can block pornographic websites, which hypothetically increases the number of barriers to accessing pornography, giving the user time to resist their urges better. However, these apps can often be turned off or bypassed and may not block the entire spectrum of sexual content, such as audio or text. Progress has been made on the use of machine learning models to detect pornographic imagery automatically, and these models may be incorporated into this kind of software in future [19]. However, these systems need to be used with care because the potential for false positives is high, leading to censorship of harmless content such as tutorials on sexual health [20], which could ultimately be harmful. Such a filter must be sensitive enough to capture a substantively high percentage of pornographic material but not so sensitive that it makes internet browsing a frustrating experience because of too many false positives, which can cause users to uninstall the program; for instance, biasing the software toward a skin-based detection method can lead to false positives in many domains (such as swimming, wrestling, or underwear modeling) and false negatives for sexual content of a nonnude nature [19]. This classification problem will also require constant updating as new sexual content and imagery forms are produced. In addition, these classifiers often do not recognize alternative media such as erotic literature or audio.

Browser extensions that provide broad-spectrum content blocking, such as uBlock Origin [21] and BlockSite [22], can also be configured to block pornography. Many of these digital self-control tools (DSCTs) are free (or have a free tier) and are thus available and effective for a broad consumer base [23]. These DSCTs are also examples of a just-in-time adaptive intervention (JITAI), where the intervention is delivered when the user needs it most—as they are beginning down the path toward relapse by browsing a website that they know to be tempting [24]. An example of a scientifically grounded JITAI is the Addiction–Comprehensive Health Enhancement Support System, a smartphone app designed to improve continuing care for alcohol use disorders [25]. However, there seems to be no evidence-based version of this for PPU.

### Interventional Studies

Content-blocking apps are just 1 example of a wide variety of tools available to help people with PPU. However, very little research has examined the effectiveness of any of these tools. Although some studies have examined the efficacy of short interventions to reduce PPU, these have generally been small scale and focused on in-person cognitive behavioral therapy (CBT) and acceptance and commitment therapy, rather than mHealth [26-29]. At the time of writing, only 1 large-scale study has investigated the effectiveness of a web-based intervention for people struggling with PPU [26]. The *Hands-off* trial, currently ongoing, is a 2-armed randomized controlled trial examining the effectiveness of a 6-week web-based PPU intervention [26,30]. The intervention arm draws from techniques such as motivational therapy, CBT, and mindfulness to provide 6 modules to the participant over 6 weeks, with baseline and follow-up surveys used to measure self-reported scores on the Problematic Pornography Consumption Scale, along with other measurements such as frequency and duration of pornography use as well as mood tracking [26,30]. Preliminary analysis shows that participants in the intervention group reported lower levels of PPU use, including frequency of use, whereas the control group showed no change in use [26]. However, the *Hands-off* intervention only assesses the effectiveness of a subset of potential features for PPU interventions and thus can only enhance the credibility of a few apps with comparable features. Furthermore, no research exists that can provide an overview of the techniques that are currently being used by consumers for PPU management.

Thus, this research aimed to categorize the features of currently available smartphone apps for managing PPU to obtain an overview of the techniques that are most prevalent and in demand. This will help to direct future research in this area and create potential therapies that can use the combined strengths of in-demand features that have been scientifically validated.

## Methods

### Feature Analysis

We performed a restricted systematic feature analysis of mobile apps available on the two leading mobile app stores: Google Play Store (Android) and Apple App Store (iOS). Apps available on either mobile phones or tablets were included in the review. The review methodology was based loosely on that used in the study by Shen et al [31], but this is not an exhaustive systematic review of all software; rather, it is an assessment of what is currently available in the mobile app space only.

On February 3, 2022, the key phrase “pornography addiction” was entered into the search bar of the 2 main mobile app stores. The results were categorized by a member of the authorship team (NH) according to a coding scheme that was iteratively updated throughout the categorization process (Textbox 1). This process was performed under the supervision of the other members of the authorship team (LD, MW, and MP). Modifications to the procedure were made when unique, nonclassifiable features were observed, in which case an adjacent category was expanded to include the feature, or a new category was created. The final coding scheme contained 16 app feature categories (Textbox 1).

**Final coding scheme used to categorize app features.**

**Feature name and type of feature**

- Track: variable tracking
- Stats: statistical insights derived from variable trackers or streak trackers
- Tutor: tutorials or coaching sessions, often delivered in written, audio, or video format
- Exercise: exercises or meditations, often in the form of cognitive behavioral therapy, motivational therapy, or hypnosis
- Block: content blocking or monitoring
- Streak: tracking of streak length (the time since the last relapse), often called a day counter, streak timer, or progress tracker
- Account: accountability partner or access to a community forum
- Diary: diary or journal with the ability to take notes or set reminders
- Badge: badges, often as rewards for reaching a new streak length
- Distract: distractions for users with high urge levels, often in the form of games, soothing music, or relaxing scenery
- Quote: motivational quotes, often from famous and historical figures
- Finance: financial tracker providing an indication of money saved via abstention from pornography use
- Locate: location tracker
- Panic: panic button, often sending the user to a site with motivational quotes, encouraging videos, or blog posts
- Religion: an explicitly religious element that is conservatively opposed to problematic pornography use
- Test: a survey that screens for pornography addiction

### Inclusion and Exclusion Criteria

Apps were screened and included in the study based on the app’s description, title, and screenshots. Only apps described as helpful for reducing pornography use were included in the review, and this included apps that had a broader focus but still mentioned pornography; for example, an app designed to target *internet addiction*, *gaming addiction*, and *pornography addiction* would have been included. Apps were excluded if they did not provide sufficient information to classify the app’s functions, offered pornographic content or did not claim to help participants to reduce or manage pornography use. Apps that were apparent duplicates of higher ranked apps as determined by their near-identical descriptions or user interfaces were recorded and excluded from further analysis.

The following information was extracted from the store descriptions of the apps: app name, creator, number of user ratings, mean rating score, and number of installations (Android only). There were several notable differences between the available information in Apple’s App Store and the Google Play Store (Textbox 2), making it challenging to aggregate statistics between the two stores.

**The differences between the available information in Apple’s App Store and the Google Play Store.**

**Notable differences between the App Store and Google Play Store with regard to app information**

- The number of app installations was only provided in the Google Play Store.
- The number and value of ratings were only recorded for the most recent version of iOS apps, whereas they were recorded for all historical versions of Android apps. This reduced the number of ratings available for iOS apps but also introduced a potential bias toward previous versions of Android apps that did not contain newer features.
- Although some apps were identical between the stores, their creator was listed under a different name for each store. Hence, best judgment had to be used to categorize identical apps between the stores.
- The Apple App Store provides specific details about in-app purchases, whereas the Google Play Store offers a range of potential in-app costs. However, the variety of pricing models for each app made the cost of apps a difficult metric to assess objectively; hence, this was excluded from the analysis.

## Results

### General Characteristics

**Figure 1:**
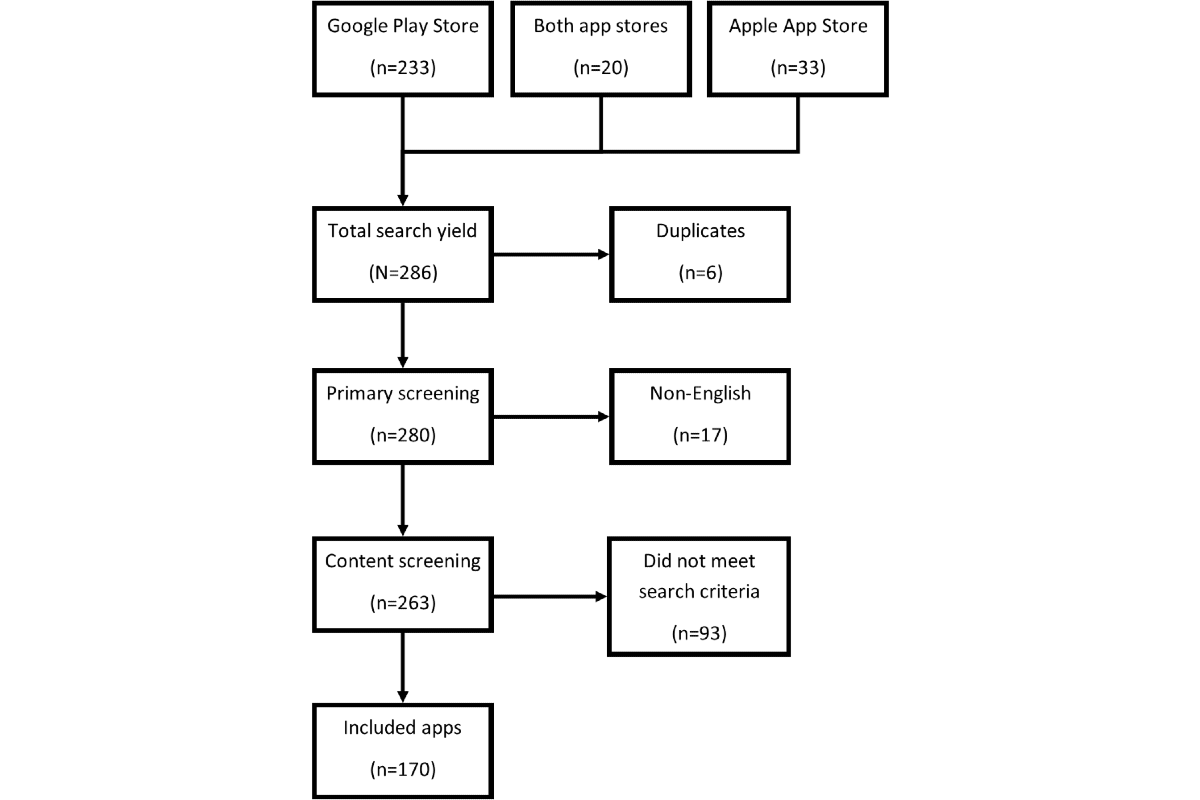
Flow diagram illustrating exclusion of apps at various stages of the study.

The data set for the content analysis can be found in Multimedia Appendix 1. The search yielded 286 apps across both platforms, of which 17 (5.9%) apps had descriptions in languages other than English and were excluded from further analysis. Of the remaining 269 apps, 93 (34.6%) were excluded because they did not aim to manage PPU, whereas 6 (2.2%) were excluded because they were duplicating an app higher in the search rankings. This left 170 relevant English-language apps to analyze, of which 121 (71.2%) were for Android devices only, and 29 (17.1%) were for iOS devices only, whereas 20 (11.8%) apps were found on both platforms (Figure 1).

Only the Google Play Store (Android) reported the number of app installations and only in predefined ranges. The most frequent range of installations for Android apps was 10,000 to 50,000, achieved by 25.6% (31/121) of the apps, whereas 2.5% (3/121) of the apps were installed in the range of 5 million to 10 million, the highest recorded range.

### Breakdown by Feature

**Figure 2:**
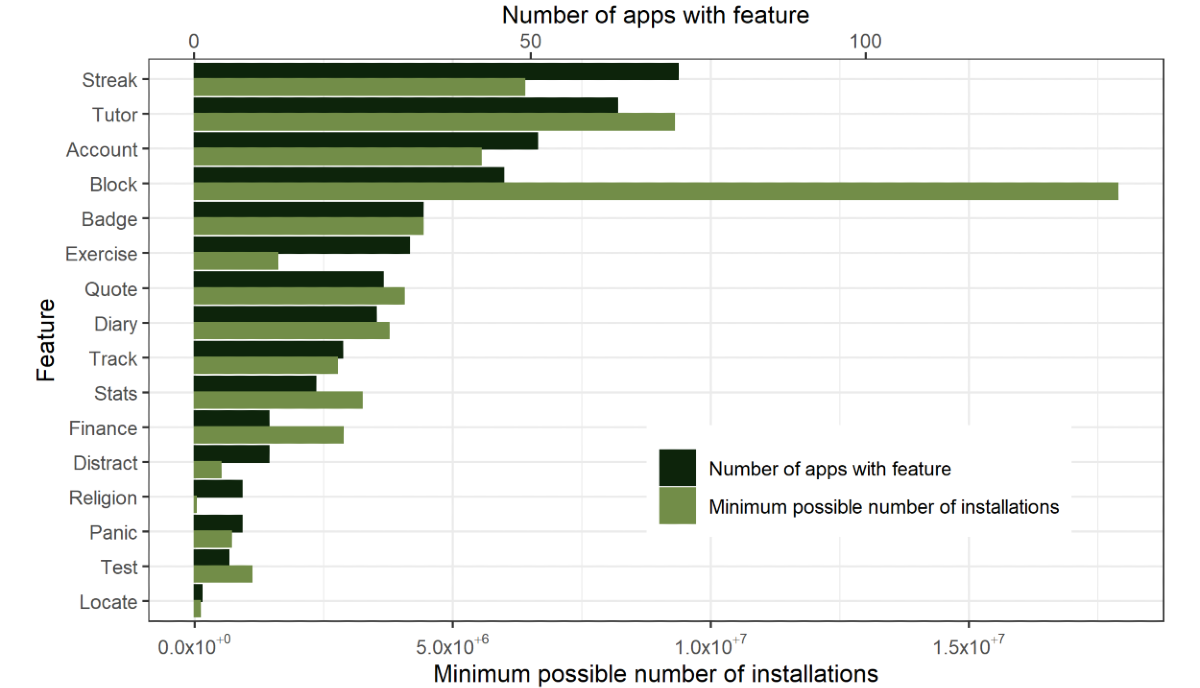
Number of apps containing each feature for both Android and iOS, and minimum possible number of installations for each feature (Android only).

The number of apps with each feature from both stores was tallied (Figure 2). The *streak* feature, which tracks the time since the last relapse, was the most common in terms of app numbers, with 42.4% (72/170) of the apps providing this feature. The *tutor* feature, providing tutorials and coaching for users, was the second most common feature, offered by 37.1% (63/170) of the apps.

In addition, it was possible to calculate the minimum possible number of installations for the Android apps with each feature by summing the minimum value in the range of installations for each app containing the feature (Figure 2). The four most common features in terms of app number (*streak*, *tutor*, *account*, and *block*) were also the four most popular features in terms of downloads, although their rank changed slightly. Notably, the *block* feature, which provided content blocking and monitoring, was only included as a feature on 27.1% (46/170) of the apps but had the highest minimum number of installations at 17,883,000, almost double that of the next highest-ranked feature (*tutor* with 9,286,505 installations).

**Figure 3:**
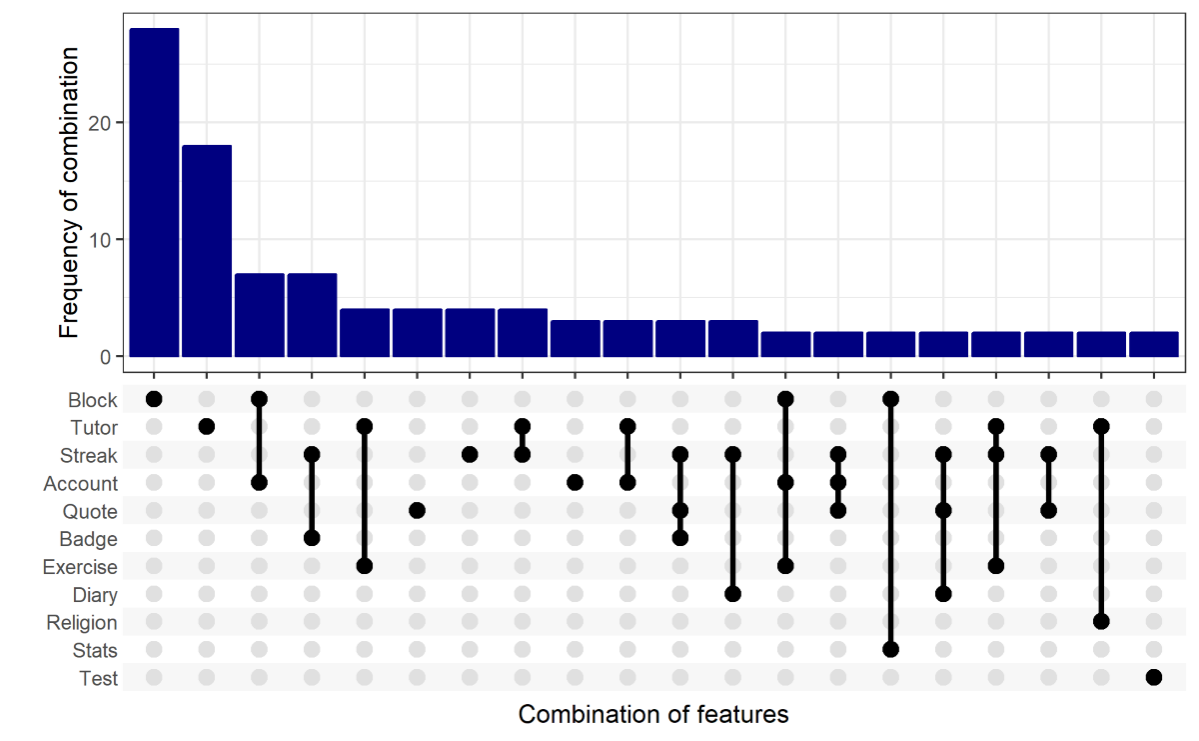
Combinatorial plot showing the most common combinations of features. Only the top 20 combinations are shown.

A combinatorial analysis was also performed. The frequency of different combinations of features was plotted to determine the most common combinations, both in terms of app frequency and the minimum possible number of installations. The results can be seen in Figure 3 for the 20 most frequent feature combinations. Of note, the two top feature combinations were single features—*block* and *tutor*—both in terms of the number of apps with only these features (28/170, 16.5%, and 18/170, 10.6%, respectively) and in terms of the minimum possible number of installations (16,002,000 and 7,244,205, respectively). The following 3 most popular combinations of features (based on minimum possible number of installations) were all individual apps: BlockerX used the *block*, *account*, *diary*, and *test* features; Quitzilla used the *stats*, *streak*, *diary*, *badge*, *quote*, and *finance* features; and I Am Sober used the *track*, *streak*, *account*, *quote*, and *finance* features.

**Figure 4:**
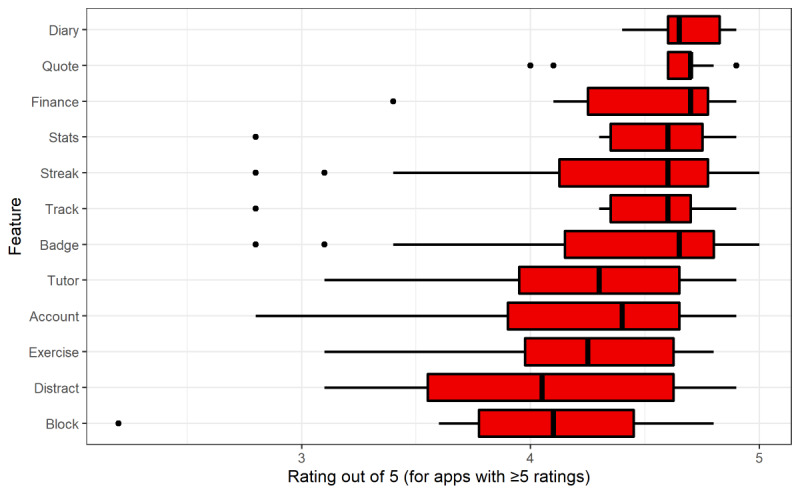
Distribution of ratings for apps with each feature, for Android and iOS, ordered by mean rating. Only apps that had received at least five ratings were recorded for each feature, and only features with at least five eligible app ratings were plotted.

App ratings out of 5 were aggregated between both stores and grouped by feature. The distributions of these ratings are summarized in Figure 4, ordered by mean rating. Only apps that had received at least five ratings were recorded for each feature, and only features with at least five eligible app ratings were plotted. None of the 4 most common features remained in the top 4 highest-rated apps by mean or median rating; *streak* came fifth on both counts, with a mean rating of 4.34 and median rating of 4.60. The *quote* and *finance* features, providing motivational quotes and financial tracking, respectively, had the equal highest median rating of 4.70, with *diary*, the journaling feature, having the highest mean rating of 4.69. *Distract*, a feature consisting of distracting activities, had the lowest median rating (4.00). *Block* had the lowest mean rating (4.04) and second-lowest median rating (4.10), despite having the highest minimum possible number of installations. *Tutor* and *account* (a feature providing an accountability partner), both of which scored highly in the minimum possible number of installations and commonality, were either eighth or ninth in the mean and median rating scores (means of 4.24 and 4.21, respectively, and medians of 4.30 and 4.40, respectively). This may indicate that features in higher demand are held to heightened scrutiny or are more challenging to implement at a high standard.

It should be noted that the ratings shown in Figure 4 only consist of apps that received enough ratings to be included in the results. Hence, there may be some bias toward superior-quality apps, as recognized by the market population.

### Review of Features Based on Installations

Of the 170 included apps, 46 (27.1%) provided the *block* feature, which provided content blocking or monitoring. Going by the minimum-possible-number-of-installations metric, this feature was the most popular by a large margin (with a score of 16,002,000). Most of this score was due to apps such as BlockSite, which has >5,000,000 downloads on the Google Play Store alone. Of these 46 apps, 15 (32.6%) provided content blocking associated with the *account* feature, usually providing an accountability partner. An example of an app with only these 2 features was Truple, which has been downloaded >100,000 times on the Google Play Store alone. This combination of features was the third most common, with 4.1% (7/170) of the apps having this combination. Still, in the ranking of the minimum possible number of installations, this combination only came 12th with a score of 270,000. However, when combined with other features, the minimum possible number of installations increased to 1,780,000. Most of this score came from the BlockerX app, which has >1,000,000 downloads on the Google Play Store alone.

Of the 170 included apps, 63 (37.1%) provided the *tutor* feature, which offered tutorials or coaching in written, audio, or video format; for example, the Life After Pornography Coach app offers >80 video lessons to aid users in their journey. However, for many apps, tutorials were not necessarily the app’s main feature but were offered as part of a complete feature set.

Of the 170 included apps, 32 (18.8%) provided the *exercise* feature, with exercises such as guided meditations or modules for CBT and motivational therapy. This feature was often combined with the *tutor* feature; for instance, an audio recording often accompanied guided meditations to direct the user through the meditation. Furthermore, 6.5% (11/170) of the apps provided the *distract* feature, often in the form of games or soothing music for users who were feeling urges.

Of the 170 included apps, 51 (30%) provided access to the *account* feature, either in the form of a web-based forum where users could discuss their PPU with others or the ability to communicate with an accountability partner; for example, users were often redirected to subreddit forums such as r/NoFap (which encourages abstinence from pornography, masturbation, and orgasm for 90 days; the name comes from the slang term “fap,” referring to masturbation) or r/pornfree (which encourages abstinence from pornography-induced masturbation but not masturbation itself).

Of the 170 included apps, 72 (42.4%) provided the *streak* feature, allowing users to track their *streak length*: the time (usually in days) since their most recent lapse. This feature was often paired with the *badge* feature, giving the user extra motivation to reach their next target or badge, and also with the *stats* feature, which provided statistical feedback on the user’s streak history and associated metrics.

Of the 170 included apps, 28 (16.5%) provided the *quote* feature in the form of motivational quotes, usually as a secondary feature of a more extensive program. Associated with this, 4% (7/170) of the apps had the *panic* feature, generally in the form of a *panic button*, which the user can press when their urge levels escalate. This typically directed a user to a website with randomized links, often with motivational quotes or a suggestion to perform a quick workout, such as 20 push-ups. An example of this is the panic button created by the r/NoFap subreddit community.

Of the 170 included apps, 27 (15.9%) apps provided the *diary* feature, allowing users to record self-reflective notes on their recovery process. This helps users to identify triggers, relapse pathways, and successful strategies for overcoming PPU.

Of the 170 included apps, 22 (12.9%) provided the *track* feature, allowing users to record their variables on a daily or instantaneous basis. Of these 22 apps, 10 (45.5%) also provided the *stats* feature, generally as a summary of insights derived from these tracked variables, often in association with the *streak* feature. Of the 170 included apps, 11 (6.5%) also included the *finance* feature, revealing how much money the user was potentially saving by reducing their pornography use.

The three least popular features were *test*, *locate*, and *religion*. Only 2.9% (5/170) of the apps provided any form of survey or test for PPU, whereas 0.6% (1/170) of the apps provided *locate* as a feature. This feature represents a type of relapse prediction service, in that the aim is to learn and highlight locations where the user may be more prone to relapse. Only 4.1% (7/170) of the apps had the *religion* feature, which had the lowest minimum possible number of installations of all features listed, with a score of 21,110. Of these 7 apps, 6 (86%) also provided the *tutor* feature, generally providing helpful scriptures, devotional passages, or prayers.

## Discussion

### Principal Findings

It seems that the academic literature on PPU is divorced from the technological interventions currently available. None of the apps reviewed contained references to literature that provided direct evidence for the app’s efficacy or safety. However, several apps reported anecdotal evidence that supported their real-world effectiveness. Furthermore, a lack of scientific studies examining a feature’s effectiveness does not necessarily mean that there is no rationale for specific in-app features. The perceived utility of a feature can be partially inferred from its demand in the real world, even if the implementation is imperfect. The relationship between feature utility and real-world demand may be mediated through the number of positive reviews left on apps with the feature, which is indicative of the feature’s effectiveness and likely to encourage a higher number of installations for these apps. Still, further research is required to consolidate the evidence base into a white list of safe apps for public consumption.

### Utility of Features

On the basis of the minimum possible number of installations, content-blocking apps are the most popular on the Google Play Store but have a lower mean rating than apps that do not offer content blocking. The high popularity score suggests that many users attempt to distance themselves from sources of pornography while trying to remain connected to smartphones and the internet. There is much anecdotal evidence on the internet supporting these apps, which are also popular in related fields such as internet addiction. Notably, Lyngs et al [32] performed a systematic review of DSCTs and found that the most prevalent feature (found in 74% of the tools) involved blocking or removing distractions. Still, despite the robust demand for these apps and their apparent utility, there are no studies investigating the effectiveness of content-blocking apps with regard to PPU reduction. However, the comparatively low mean rating suggests that these apps are not as effective as their users would hope, compared with their expectations for other features.

It is possible that these content-blocking apps may be advertised more than apps with other features, and customers may resent paying for these apps, which are generally more expensive than other apps because of technical requirements such as virtual private network services. By contrast, as discussed earlier, it is challenging to create and maintain a content filter with sufficient sensitivity and specificity to satisfy the market mainly because web-based content keeps evolving. The mismatch between the difficulty of this task and the expected quality of these apps may be more significant than the difficulty-quality mismatch for other features; for instance, an app that provides CBT tutorials may only need to provide minimal benefit for its users to rate it highly because the user will not blame the app for causing a relapse. However, if a content-blocking app fails to block a new porn site, which leads to a user relapsing, this will likely lead to a negative review. This may explain why the *block* feature scored very highly in terms of the minimum possible number of installations but had a relatively low median rating compared with other features.

An interesting alternative to counteract this weakness is Truple, an app that takes screenshots of all browsing behavior, analyses them, and flags potential sexual content to an accountability partner [33]. In theory, this reduces the temptation to uninstall or bypass the app, which is a problem for some apps with the *block* feature. However, as is the case with using a therapist, not all users are willing to share their private browsing information with even trusted friends, which may reduce use of this feature. In addition, the app does not prevent users from viewing the websites, which indicates that it may be fortified by the addition of the *block* and *quote* features. Motivational quotes have some potential to provide positive boosts to self-esteem and self-efficacy for patients using mHealth interventions to recover from PPU [34]. The addition of motivational quotes may provide timely reminders to stay away from distracting material that has the potential to devolve into PPU.

Badges seem to have potential as a means of gamifying the recovery process and generating positive reinforcement [35] and have been shown to improve motivation in therapeutically administered tasks [36]. However, the use of progress trackers is controversial. Despite being popular, it is plausible they can be detrimental to users who relapse because they may induce additional feelings of guilt and anxiety upon breaking a streak, perpetuating the feelings that led to a relapse in the first place [31]. Still, these feelings may be a necessary part of the recovery process. Using the prospect theory framework developed by Kahneman and Tversky [37], we can define the risk of experiencing postrelapse depression as a potential loss, which the user must compare with the momentary pleasure of relapse as a gain. In this framework, healthy use of the *streak* feature creates a positive feedback loop, whereby the user experiences increasing loss aversion as their streak length increases, and memories of past failures inspire greater motivation to reduce future losses. However, an alternative positive feedback loop is also possible, where the feelings of guilt and depression brought about by relapse cause the user to turn to pornography to avoid these feelings [38]. These questions invite further research to determine whether the utility of this feature varies within a population of participants struggling with PPU.

In many ways, apps combining the *track* and *stats* features act as a self-monitored ecological momentary assessment, where the user both records and analyses their data [39]. Such an app’s effectiveness will be inherently limited by the ability of its users to interpret such data accurately, particularly those users without a sufficient level of scientific training. Hence, app creators must balance the complexity of the statistical analysis they provide against the ability of their users to interpret these data meaningfully.

The Fortify app (as part of Impact Suite) allows users to track their variables over time, including urges and variables related to well-being. It then performs analytics on these variables over time, allowing users to self-examine their weaknesses. The app also includes a personal journal, training and meditation sessions, community forums, and accountability partners. This is the only mobile app we found with a comparable alternative in the literature, although that trial (the *Hands-off* study) is not yet complete and focuses more on providing coaching modules and exercises for users [30]. However, it has shown promising results so far, with indications that the combination of variable tracking with modules and community messaging are an effective way to manage PPU [26].

Although several apps provide the ability to record one’s urges at the end of each day, we found no app that allows the user to record their urges instantaneously, with associated follow-up recommendations such as taking a break from the internet or performing exercise. If a user performs urge tracking at regular intervals, they can monitor responses to various stimuli in real time and adjust their behaviors when urge levels increase; for example, a user may find that their urges increase rapidly while browsing a particular website and choose to regulate their exposure to this website. In addition, once they have been tracking their urges for a while, the user may become more attuned to scenarios that increase their urges and formulate strategies to escape these scenarios. This could take the form of a self-regulated JITAI, where the user records their urge levels and responds to the app’s subsequent prompt to change their environment, perform exercise, or meditate.

### Motivational Readiness

Despite their lack of grounding in the literature, many of the apps in this analysis seem to have features that align with addiction-management frameworks such as self-regulation theory [40] and self-efficacy theory [41]. However, one theory allows us to quantify the game-theoretic effectiveness of the features seen in this review: the dual-threshold model of motivational readiness developed by Kruglanski [42,43]. In this theory, the degree to which a person pursues a goal state (such as gratification of pornographic urges) is a function of both their motivational readiness to achieve the state and their belief that the state can be achieved (expectancy) [42]. To put this in the context of PPU, if the user believes that they can access pornography (expectancy), and their desire for pornography is stronger than their will to reduce PPU (motivational readiness), then they will take steps to use pornography in proportion to their desire to use it. However, if they do not believe that they can access pornography, then they will not attempt to use pornography, irrespective of their desire to do so.

On the basis of this model, we propose two potential strategies to reduce PPU: either raise the person’s motivation to reduce PPU (strategy 1) or increase barriers to pornography access (strategy 2). From a game theory perspective, strategy 2 is superior to strategy 1 because if the user does not meet the requisite threshold for expectancy, they cannot take action to use pornography, regardless of their motivation to do so.

If we try to categorize the features presented in Textbox 1 based on these strategies, only two features, *block* and *locate*, clearly fall under strategy 1, whereas the other features all fall under strategy 2 (excluding *test*, which is for assessment only). The *locate* feature has a fundamental flaw, in that users regularly spend time in what the feature would consider a high-risk location for pornography use: their own bedroom. Hence, *block* is perhaps the only feature with the potential to eliminate PPU entirely by simply removing the ability to view pornography. Of course, it is virtually impossible to cut off all sources of pornography in the postinternet age. However, the hypothetical superiority of strategy 2 may explain the popularity of content-blocking apps in this paper’s analysis.

### Defense in Depth, or the Swiss Cheese Model

Given that both these strategies have their weaknesses, a potential user may consider combining the strategies to reduce PPU; for example, they may use a *block* app in conjunction with a *track* app to simultaneously monitor their urge levels, place barriers between themselves and exposure, and predict times and environments where they will be at higher risk of relapse. If the *exercise* and *tutor* features with training modules and CBT elements are added, the user would have tools available to both increase their motivation and reduce their expectancy.

This combining of techniques is an example of the *defense in depth* (DiD), or *Swiss cheese* model of risk management, applied to PPU. DiD is a concept created by the National Security Agency to harden their computer security. Multiple layers of defense are placed within a DiD system to provide redundancy of protection if a vulnerability is uncovered [44]. Applying this model to PPU, one could view pornography as a *virus* targeted at the brain. If the user has not put up enough layers of *security*, then the virus will *infiltrate* the mind and cause a relapse. Using one layer of security such as the *block* feature will provide some protection but leave multiple vulnerabilities open for the virus to penetrate, much like the holes in a slice of Swiss cheese. However, combining multiple layers of security (for instance, adding regular CBT to the user’s routines) will reduce the probability of relapse even further by removing some of the *holes* left behind by prior layers of security. Having a unique feature combination may also help create a novel market niche, which seems to have been a successful strategy for several apps.

The DiD framework may also benefit apps seeking to differentiate themselves in a competitive app market. From the combinatorics analysis performed in this review, it can be deduced that there are two main strategies implemented by the most successful apps in this space: (1) either implement one desirable feature (such as *block*) exceptionally well, or (2) create a niche market by combining a unique set of features. The second strategy, which aligns with the DiD framework, has the potential upside of avoiding competition from numerous other apps, which is the challenge facing apps that only use either the *block* feature or the *tutor* feature.

Even when combined, these technologies are likely not entirely sufficient. People who struggle with compulsive behaviors tend to search for new pathways to indulging in their behavior and may find ways to bypass multiple protective layers, mainly when triggered or under stress [23]. Hence, the defense set up by the user should comprise both static layers that are always present and dynamic layers that are updated regularly to adjust for any blind spots that may appear [44]. Using this framework, an example of a dynamic layer would be using the *track* feature to identify new triggers, whereas an example of a static layer would be using pornography blocker software on a web browser. Most users trying to overcome PPU will find that complete abstinence is difficult, but by using the DiD model, they can treat each relapse as a weakness to be fixed in their quest to eliminate PPU.

In addition, the *track*, *streak*, and *stats* features can guide the user to adjust their strategy when new weaknesses are uncovered through self-analysis of their data. However, not all users will be equipped with the ability to interpret their data accurately. Furthermore, a software-generated interpretation of user data may provide some insights. Still, it will be limited in the amount of nuance it can collect, particularly when the user’s data do not match regular trends. This is where a trained therapist would prove to be a significant addition to the DiD strategy, in that they could provide insights that both the user and their software tool are unable to uncover. The therapist will also be less vulnerable to self-evaluation bias.

### Weaknesses of mHealth Solutions

The use of apps to counter apps is counterintuitive [13] but may be necessary for certain users to form new habits. One of the difficulties of providing a technological intervention for PPU is that pornography is delivered on the same technologies; for example, if one uses a mobile app to track one’s relapse statistics, one will likely be using the same device one uses to access pornography. This can have both positive and negative consequences. Some app users may be able to use the intervention to develop alternative habits. However, it is possible that for other users, the reverse will happen, where they link the intervention to continued pornography use, making it a double-edged sword. A separate device (such as a pager) is always an option, but these devices generally have limited functionality compared with a smartphone. To increase the reach of an app, highly accessible technologies should be used, which in the case of pornography users is most likely their smartphone [45].

No apps in this review had any screening tools for PPU grounded in the literature. It could be argued that a scientifically validated assessment should be mandatory for any app claiming to help with PPU management. Although there remains controversy around the assessment criteria for PPU [46], the lack of a test implies that the user has a problem by default. It could be argued that a false positive assessment of PPU can induce moral incongruence and subsequent anxiety or depression, potentially leading to a positive feedback loop that increases PPU in some users.

This also highlights that many web-based tools are most effective when guided and recommended by a qualified therapist [47]. Much work needs to be done to bring the apps reviewed in this study to the therapeutic space, especially considering the lack of therapists trained to treat PPU [48,49]. A therapist may also be able to warn against unintended consequences of certain apps; for example, several apps in this review provided distractions in the form of games or calming music. This feature could be viewed as an alternative to guided meditation, in that it offers a way to direct attention away from pornography. However, many users also struggle with internet or gaming addictions, meaning that there is a risk that the user could justify replacing PPU with addictive gaming behaviors. It is possible that the most effective apps for reducing PPU are designed so that the user no longer requires the app once they have achieved their goals and even uses their smartphone less, reducing temptation risk in general. This may be more difficult for apps with the *distract* feature if the app has an addictive design.

### Limitations

The aforementioned quantitative feature analysis focused on mobile apps and excluded other viable software; for example, several browser extensions provide a *block* feature, such as uBlock Origin [21] or BlockSite [22], that may not specifically be designed for reducing PPU but can be used to the same effect. One such app, Plucky, blocks all images and videos on the internet (besides a white list of allowed websites) and makes itself *inconvenient* both to uninstall and to edit the white list [50]. The user can customize the app but only after a predefined delay period. In theory, this provides the user with time to overcome their urges and resume baseline behavior.

There are several apps available on the internet that provide variations on the accountability feature, including X3Watch [51] and Qustodio [52], that were excluded from this review. Many of these apps are designed to broadly reduce internet use, of which pornography is a use case. Even the r/NoFap subreddit and the associated NoFap website could be considered a technological *intervention* of sorts, providing the *streak*, *account*, *tutor*, *exercise*, and *panic* features [53]. In summary, there are many self-help resources and associated software-based interventions available on the web, many of which could be used to complement the mobile apps included in this review.

Any statistical analysis of mobile app stores, including descriptive statistics, is hampered by a lack of information. Both app stores use various methods, including hiding in-app payment and advertising information and using large categories for aggregating and blurring popularity metrics. Variables are not identical between the stores and reflect the preferences of different customer bases; for instance, on average, Apple customers have greater financial resources than Android customers because Apple mobile phones tend to be more expensive than equivalent Android mobile phones [54]. In addition, it is impossible to deduce how much promotional work has been done behind the scenes to advertise these apps. Hence, the popularity metrics provided here are a poor representation of the actual demand for, and quality of, features and should only be interpreted as indicative of potential trends in this space. Non–English-language apps were also excluded, providing another potential source of bias to these results.

Furthermore, any measure of popularity is likely to be biased in cases where apps are used for purposes other than managing PPU; for example, BlockSite, which has at least 5,000,000 downloads on the Google Play Store and contains the *block* feature, is also provided to the user for *blocking distracting websites*, not just pornographic ones [22]. Removing this app and others with multiple purposes may affect the ranking of features in the popularity metrics used for this study.

### Conclusions

The mobile app space is replete with apps that seem to be beneficial to users attempting to manage PPU based on their generally high app store ratings, positive reviews, and high number of installations. In particular, content-blocking apps have clear potential for reducing PPU by removing access to pornography on the user’s device. However, there remains a substantive lack of evidence in the scientific literature to quantify the effectiveness of such apps. Although each of these apps has its purported benefits, the most effective method for reducing PPU may lie in combining the most robust features in this space, using a DiD strategy of risk management. This could be achieved by using multiple apps at once, although this should probably be performed under the guided hand of a trained therapist, if possible. However, such a strategy is inefficient and cumbersome. Further work is required in this space, both to research the effectiveness of these app features and to consolidate the evidence base into a white list of safe apps for public consumption.

## Appendix

Multimedia Appendix 1 This Microsoft Excel file contains the data set generated during the app review. It is separated into two sheets: the first contains the data set, and the second contains a data dictionary describing each variable in the data set.

## Abbreviations

CBT: cognitive behavioral therapy
DiD: defense in depth
DSCT: digital self-control tool
JITAI: just-in-time adaptive intervention
mHealth: mobile health
PPU: problematic pornography use
